# Improvement Effect of *Bifidobacterium animalis* subsp. *lactis* MH-02 in Patients Receiving Resection of Colorectal Polyps: A Randomized, Double-Blind, Placebo-Controlled Trial

**DOI:** 10.3389/fimmu.2022.940500

**Published:** 2022-06-27

**Authors:** Hui Liu, Kaige Zhang, Peng Liu, Xuan Xu, Yuyang Zhou, Lihong Gan, Ling Yao, Bin Li, Tingtao Chen, Nian Fang

**Affiliations:** ^1^ Third Clinical Medical College, Nanchang University, Nanchang, China; ^2^ Department of Gastroenterology, The First Hospital of Nanchang (The Third Affiliated Hospital of Nanchang University), Nanchang, China; ^3^ Huankui Academy, Nanchang University, Nanchang, China; ^4^ National Engineering Research Center for Bioengineering Drugs and the Technologies, Institute of Translational Medicine, Nanchang University, Nanchang, China

**Keywords:** *Bifidobacterium*, MH-02, colorectal polyps, postoperative symptoms, intestinal microbiota

## Abstract

**Background:**

Postoperative symptoms, bowel dysfunction and recurrence are common problems after resection of colorectal polyps. We aimed to evaluate the efficacy of *Bifidobacterium* in the postoperative patients.

**Methods:**

In this single-center, randomized, double-blind, placebo-controlled trial, adults (≥ 18 years) undergoing endoscopic resection of colorectal polyps were treated with probiotics (*Bifidobacterium animalis* subsp. *lactis* MH-02, 2 × 10^9^ colony-forming units per packet) or placebo once daily for 7 days. The primary clinical endpoint was a reduction in the mean total postoperative symptoms score within 7 days postoperatively. Secondary clinical endpoints were the single symptom scores, time to recovery of bowel function, and changes in the intestinal microbiota. This study is registered with the number ChiCTR2100046687.

**Results:**

A total of 100 individuals were included (48 in probiotic group and 52 in placebo group). No difference was seen in the mean scores between the two groups (0.29 vs. 0.43, P = 0.246). Colorectal polyps size (P = 0.008) and preoperative symptoms (P = 0.032) were influential factors for the primary endpoint. Besides, MH-02 alleviated difficult defecation (P = 0.045), and reduced the time to recovery of bowel function (P = 0.032). High-throughput analysis showed that MH-02 can help restore the diversity of intestinal microbiota, and increased the relative abundance of *Bifidobacterium*, *Roseburia*, *Gemmiger*, *Blautia* and *Ruminococcus*, while reduced the relative abundance of *Clostridium* at genus level (P < 0.05).

**Conclusion:**

In this prospective trial, MH-02 showed efficacy in patients with resection of colorectal polyps, particularly in the recovery of bowel function, and the changes in the intestinal microbiota may provide evidence for further exploration of the therapeutic mechanisms.

## Introduction

Colorectal polyps are a common intestinal disease characterized mainly by protruding masses on the mucosal surface of the colorectum (1). It occurred more often in people over the age of 40, and the prevalence in the Chinese population is as high as about 20% (2). Although colorectal polyps are considered as benign lesions, certain specific pathological types such as adenoma can develop into colorectal cancer (3). Endoscopic resection of colorectal polyps is an early preventive measure for colorectal cancer (4), but the procedure often results in the onset of postoperative complications such as bleeding, abdominal pain and bloating (5), accompanied by a high recurrence rate (6). Thus, there is an urgent need to find agents that reduce postoperative complications and recurrence of colorectal polyps.

Intestinal microbiota is a symbiotic ecosystem containing trillions of bacteria, which plays a key role in human health (7). The dysbiosis of intestinal microbiota is strongly associated with various diseases such as inflammatory bowel disease, diabetes, chronic kidney disease, and tumor (8). A study that enrolled 780 individuals showed that the relative abundance of *Bilophila*, *Desulfovibrio* and *Mogibacterium* was significantly higher in patients with adenomatous polyps (9), suggesting a crucial role of the intestinal microbiota in the development of colorectal polyps. Resection of colorectal polyps requires a bowel preparation in which flushing of large amounts of fluid and disruption of the anaerobic environment can lead to severe alterations in the intestinal microbiota, especially the reduction of Bacteroidetes and Firmicutes (10). Moreover, injury to the intestinal mucosa during resection of multiple polyps can cause varying degrees of mucosal inflammation (11), which may exacerbate intestinal microbiota dysbiosis and lead to abdominal symptoms. In addition, it has been shown that the intestinal microbiota composition didn’t change significantly 3 months after colorectal polypectomy, and this preoperative-like intestinal ecology may be responsible for the recurrence of colorectal polyps (12). Therefore, the resection of colorectal polyps may lead to a severe imbalance of intestinal microbiota for a short period of time, which may result in the onset of symptoms such as abdominal pain and bloating, while the long-term effects are not significant.

Probiotics are live microorganisms considered to be beneficial to the host if consumed sufficiently (13). As an essential member of probiotics, *Bifidobacterium*, a Gram-positive anaerobic bacterium, has the ability to immunomodulate, inhibit pathogens, produce bacteriocins and maintain intestinal microbiota homeostasis (14). An *in vitro* experiment demonstrated that *Bifidobacterium* could inhibit the proliferation of several human colorectal cancer cell lines (15), suggesting that it has tumor suppressive effects. Our previous studies showed that oral *Bifidobacterium* reduced gastrointestinal symptoms, decreased inflammation and promoted restoration of intestinal microbiota diversity in patients after gastric cancer surgery (16). Another study showed that administration of *Bifidobacterium* after colorectal cancer surgery also reduced levels of inflammatory factors such as TNF-α, IL-6, IL-10, IL-12, IL-17A, IL-17C and IL-22 (17). Despite the significant role of *Bifidobacterium* in the prevention and adjuvant treatment of tumor, clinical trials on the effect of *Bifidobacterium* in patients receiving resection of multiple colorectal polyps have not been seen.

In this study, *Bifidobacterium animalis* subsp. *lactis* MH-02 was used to evaluate its effect on the symptoms and recovery of intestinal function in patients receiving resection of colorectal polyps, and high-throughput sequencing was performed to evaluate the effect of MH-02 on postoperative intestinal microbiota, in order to provide a scientific basis for the application of probiotics after resection of colorectal polyps.

## Materials and Methods

### Study Design and Participants

This study was a single-center, double-blind, parallel group design, placebo-controlled trial. Patients were recruited from the gastroenterology inpatient unit of the First Hospital of Nanchang. All patients included in this trial underwent high-quality bowel preparation under professional guidance into the day before the colonoscopy procedure. Adult patients (≥ 18 years) diagnosed postoperatively with multiple colorectal polyps (at least 3) and resected endoscopically met inclusion criteria. Patients who had undergone abdominal surgery, had significant malignant lesions or inflammatory bowel disease under colonoscopy, had poor general condition, or had a history of allergy to drugs or probiotics were excluded. Patients who had been taking antibiotics, immunosuppressants or probiotics for the last three months were also excluded.

All patients participating in this study signed informed consent. This study was supervised by the Ethics Committee of the First Hospital of Nanchang (No. KY2021040) and registered in the Chinese Clinical Trial Registry with the registration number ChiCTR2100046687. All surgeons had rich experience in endoscopic operation. Patient demographic data, surgical information, past medical history, postoperative symptoms, defecation and laxative use were recorded, and the largest polyp diameter and highest pathological grade were recorded for multiple polyps statistics. All clinical data collection was done at the First Hospital of Nanchang.

### Randomization and Masking

Participants were assigned 1:1 to either the probiotic group (P-Bb) or the placebo group (P-N) using random number table method by a non-participating staff member who provided the probiotics to the investigator after patient enrollment. There were no significant differences in packaging, color, or odor between probiotics and placebo, thus ensuring a double-blind status between investigator and patient. The staff member and the investigator remained masked until the end of the experiment.

### Trial Protocol

Patients who met the eligibility criteria were randomly assigned to either the probiotic group (P-Bb) or the placebo group (P-N). Patients enrolled in the group started eating (light and easily digestible food such as thin rice and crumbled noodles) at 4 hours postoperatively and were asked to take the probiotic preparation we provided continuously for 7 days postoperatively, during which spicy and stimulating diet and alcohol consumption were prohibited. The experimental probiotics was a mixture of MH-02 and maltodextrin with 2 × 10^9^ colony-forming units per packet of live bacteria. The placebo contained only the same grams of maltodextrin. MH-02 was provided by Harbin Meihua Biotechnology Co, Ltd, Harbin, Heilongjiang, PR China, and was stored in a refrigerator at 4°C. Probiotics and placebo are both taken one packet per day. Treatment compliance of patients was obtained by counting the number of pouches used, and good compliance was defined as using more than 80% after 7 days.

Patients were evaluated daily by a trained physician using a questionnaire in the postoperative period. The questionnaire included 3 common symptoms after colorectal multiple polypectomy: abdominal pain, bloating, and dyspareunia. Other symptoms such as dizziness, diarrhea, and hematochezia were not included in the analysis because of their short duration of occurrence or low incidence. The above symptoms were scored using a 4-point Likert scale (0-3, 0 = ‘symptom absent’, 1 = ‘mild ‘, 2 = ‘moderate’ and 3 = ‘severe’). The time when the patient started to experience self-initiated bowel movement, the use of laxatives and adverse reactions were also recorded. Stool samples were collected 5-7 days postoperatively in centrifuge tubes containing 30% sterilized glycerol and stored at -80°C in a refrigerator pending sequencing analysis.

### Outcomes

The primary clinical endpoint was the improvement in patients’ postoperative symptoms (including abdominal pain, bloating, and difficult defecation), as demonstrated by the reduction in the mean total postoperative symptoms scores between the P-Bb group and the P-N group within 7 days after the procedure. Patients were interviewed face-to-face or by telephone using a questionnaire, and were scored according to severity for any of these symptoms. Patients with multiple symptoms at the same time were scored in parallel.

Secondary clinical endpoints were single symptom scores, time to recovery of bowel function, the proportion of patients who had difficult defecation, and the use of laxatives. Time to recovery of bowel function was defined as the number of days since patients first experienced self-initiated bowel movement (excluding bowel movements that occurred after laxative use, which was recorded as 7 days if no self-initiated bowel movement occurred for more than 7 days) (18). The secondary biological endpoint were the changes in intestinal microbiota, as demonstrated by the difference in α­diversity, β-diversity and species composition between the two groups, as well as the analysis of the microbiota with differences in species composition compared to normal subjects.

### DNA Extraction and High-Throughput Sequencing

Methods were provided by the technicians at Personal Biotechnology, Co., Ltd. (Shanghai, China). DNA kits were used to extract the bacterial DNA from the collected stool samples. The primer sets 338F (5′- ACTCCTACGGGAGGCAGCA -3′) and 806R (5′- CGGACTACHVGGGTWTCTAAT -3′) were used to amplify the hypervariable V3V4 region of the 16S rRNA gene. The PCR-amplified products were double-ended sequenced using the Illumina MiSeq platform. ASV/OTU signature sequences were obtained using the DADA2 method, followed by processing using Quantitative Insights into Microbial Ecology (QIIME). The taxonomic classification was performed using the Greengenes database v13.8 (19). The samples we collected (group P-Bb, P-N) were analyzed for α-diversity, β-diversity, and species differences. We also collected high-throughput sequencing results of 20 healthy subjects (group C) in the NCBI public database (PRJNA706061) and performed species composition analysis with both P-Bb and P-N. Group C was described as preoperatively collected stools based on the literature, and was confirmed as a healthy population by colonoscopy (20).

### Data Analysis

Since there is no authoritative published analysis of the efficacy of MH-02 applied after resection of multiple colorectal polyps, it is impossible to make a reasonable power analysis. Based on a previous study (21), we assumed that the mean total postoperative symptom score was 1.00 for the P-Bb group and 0.70 for the P-N group, with a standard deviation of 0.5 in both groups, we needed to enroll 44 people in each group for study (power of 80% and α = 0·05). Considering a 10% dropout rate, we ultimately planned to enroll 48 people in each group.

Patients were analyzed based on intentional analysis for the final analysis. Missing data were imputed with last observation carried forward. Data were analyzed or charted by GraphPad Prism (v8.0) and SPSS (v22.0). Quantitative data are shown as mean ± standard deviation or median (interquartile range), and qualitative data are expressed as rates. The mean score of all symptoms and the mean score of each symptom after the procedure were analyzed by using multiple regression analysis. Covariates or factors were selected from gender, age, polyps size, number, location, technique type, and preoperative symptoms. Other outcomes were analyzed by unpaired t-test for quantitative data and Fisher’s exact test or chi-square test for qualitative data. Two-sided P < 0.05 was used as the basis for significant differences. Since probiotics were defined as foods rather than drugs, no Data Monitoring Committee (DMC) for clinical trials was used.

## Results

**Figure 1:**
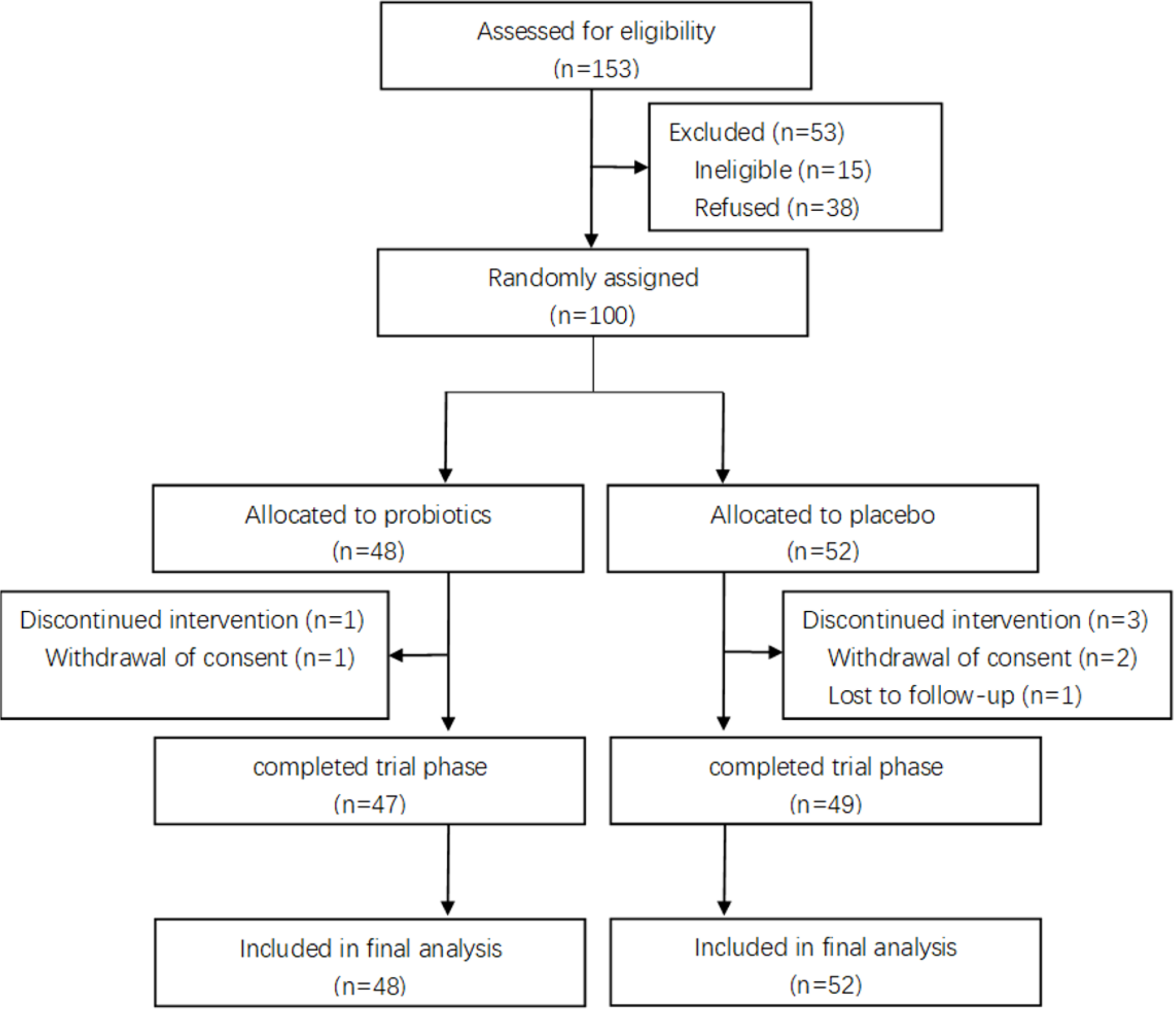
Flowchart of the trial.

From June 01, 2021 to October 31, 2021, a total of 153 individuals were assessed for eligibility, with 100 individuals randomly assigned and included in the final analysis, 48 in the group P-Bb and 52 in the group P-N. During the study, 1 participant in group P-Bb failed to complete the full study due to withdrawal of consent while 3 participants in group P-N (2 withdrawal of consent, 1 lost to follow-up) (**Figure 1**). Baseline information (**Table 1**) showed no significant differences between the two groups of patients in terms of age, gender, BMI, polyps data and technique types.

**Table 1:** Baseline characteristics.

	P-Bb (n = 48)	P-N (n = 52)	*P*-value
Age (y) (mean ± SD)	58.67 ± 9.44	59.25 ± 11.33	0.781
Sex (female, n [%])	18 (37.50)	22 (42.31)	0.624
BMI (mean ± SD)	23.84 ± 3.33	24.03 ± 3.04	0.756
Size (mean ± SD)	0.61 ± 0.33	0.64 ± 0.34	0.614
Number (n [%])			0.858
3-9	43 (89.58)	46 (88.46)	
≥10	5 (10.42)	6 (11.54)	
Localization (n [%])			0.653
Single-site	10 (20.83)	9 (17.31)	
Multi-site	38 (79.17)	43 (82.69)	
Histology (n [%])			0.653
Inflammatory	1 (0.02)	3 (0.06)	
Hyperplastic	18 (0.38)	23 (0.44)	
Adenomatous	27 (0.56)	24 (0.46)	
Other	2 (0.04)	2 (0.04)	
Technique (n [%])			0.999
APC/EMR	46 (95.83)	49 (94.23)	
ESD	2 (4.17)	3 (5.77)	

Among the 100 participants included in the analysis, 74 participants had preoperative symptoms such as abdominal pain, bloating, abnormal bowel habits or others (33 in group P-Bb and 35 in group P-N), and 26 participants had no discomfort (**Supplementary Table 1**). The result of multiple regression analysis of the primary endpoint showed that there was no significant difference between the two groups in the mean total postoperative symptoms score (P = 0.246). Meanwhile, there were statistical differences in the effects of polyps size (b = 0.57, t = 2.71, P = 0.008) and preoperative symptoms (b = 0.30, t = 2.18, P = 0.032) on the primary endpoint. Statistical analysis of individual symptom score using this analytical model showed a difference between the two groups only for the symptom of difficult defecation (P = 0.045), while no difference was seen in abdominal pain and bloating (**Table 2**). In addition, there was a statistically significant difference in the days to first self-initiated bowel movement (3.62 versus 2.90, P = 0.032). More people in the P-N group had difficult defecation than in the P-Bb group and required the use of laxatives more frequently during the consultation (P = 0.032) (**Table 3**).

**Table 2:** Postoperative symptoms.

Mean score - median (interquartile range)	P-Bb (n = 48)	P-N (n = 52)	*P*-value^*^
Total	0.29 (0.00-0.68)	0.43 (0.00-1.00)	0.246
Pain	0.00 (0.00-0.00)	0.00 (0.00-0.29)	0.968
Bloating	0.00 (0.00-0.29)	0.21 (0.00-0.57)	0.364
Difficult defecation	0.00 (0.00-0.00)	0.00 (0.00-0.00)	0.045

^*^ All P- values are from a multiple regression analyses adjusted for the variables as the primary outcome.

**Table 3:** Postoperative bowel function.

	P-Bb (n = 48)	P-N (n = 52)	*P*-value
Time to recovery of bowel function (mean ± SD)	2.90 ± 1.39	3.62 ± 1.87	0.032
Difficult defecation (n [%])	4 (0.08)	12 (0.23)	0.057
Laxative use (n [%])	1 (0.02)	8 (0.15)	0.032

**Figure 2:**
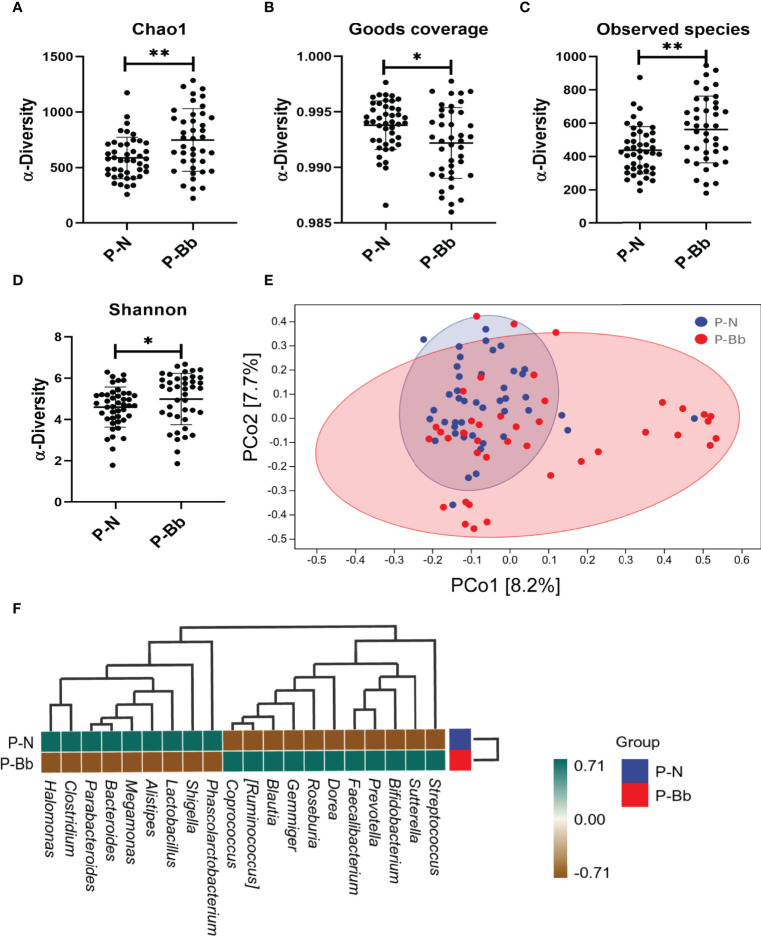
MH-02 had an improvement effect on postoperative intestinal microbiota. Values are presented as means ± SD (41 P-Bb, 44 P-N). **(A)** The Chao1 index. **(B)** The Goods coverage index. **(C)** The Observed species index. **(D)** The Shannon index. **(E)** PCoA of β-diversity index. **(F)** The clustered heat map of P-Bb and P-N. *p < 0.05, **p < 0.01.

Finally, 85 fecal samples (41 P-Bb, 44 P-N) were collected. In α-diversity, the two groups were significantly different in Chao1 (P < 0.01) (**Figure 2A**), Observed species (P < 0.01) (**Figure 2C**), Shannon (P < 0.05) (**Figure 2D**). Goods coverage (**Figure 2B**) were approximately 1 for both groups. In β-diversity, the principal coordinates analysis (PCoA) (**Figure 2E**) exhibited that the microbial diversity in P-Bb group and P-N group were different. For the two groups, a clustered heat map (**Figure 2F**) was plotted by correlation of the top 20 intestinal bacteria of average abundance at the genus level, showing a higher relative abundance of some beneficial bacteria in the P-Bb group, such as *Bifidobacterium*, *Faecalibacterium*, *Dorea*, *Roseburia*, *Gemmiger Blautia*, and *Ruminococcus*. Among them, *Dorea*, *Roseburia*, *Gemmiger*, *Blautia*, and *Ruminococcus* were at the same taxonomic level in the clustering tree. The relative abundance of *Megamonas*, and *Clostridium* was higher in the P-N group.

**Figure 3:**
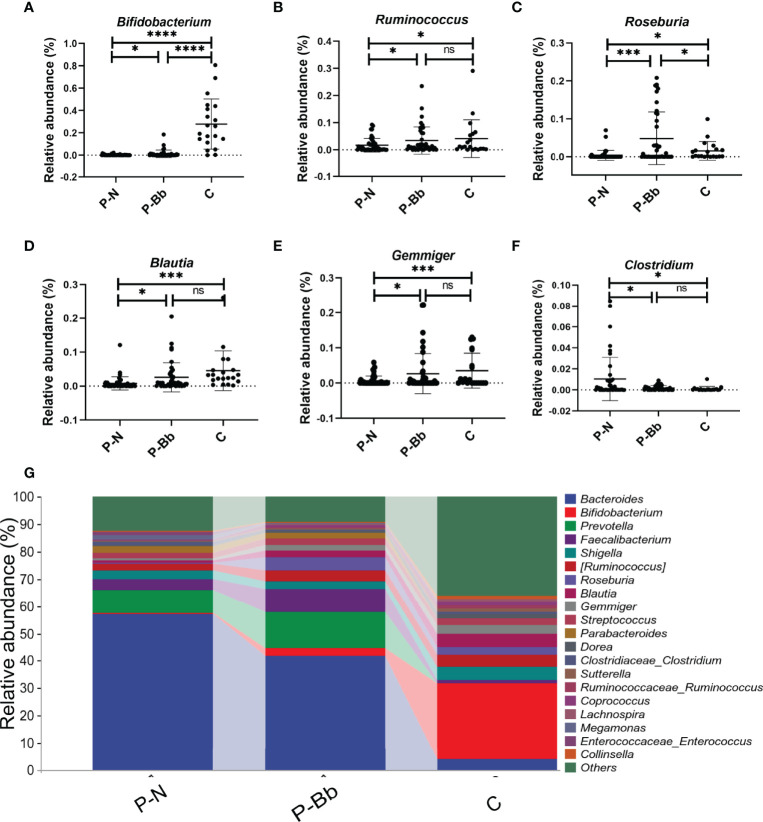
MH-02 can help restore the majority of the postoperative intestinal microbiota towards healthy people. Values are presented as means ± SD (41 P-Bb, 44 P-N,20 C). **(A–F)** The relative abundance of *Bifidobacterium*, *Ruminococcus*, *Roseburia*, *Blautia*, *Gemmiger* and *Clostridium*. **(G)** The species composition analysis of P-Bb, P-N and C group. *p < 0.05, ***p < 0.001, ****p < 0.0001, ns, no significant.

When the two groups were compared with the healthy group (C) (**Figure 3G**), it was found that the taxonomic composition of the P-Bb group and P-N group differed significantly from the C group at the genus level. The relative abundance of *Bifidobacterium* was significantly lower in both the P-N and P-Bb groups than in the C group, but higher in the P-Bb group than in the P-N group (**Figure 3A**). Compared to C group, the relative abundance of *Ruminococcus*, *Blautia*, and *Gemmiger* was significantly reduced in P-N group, while P-Bb group was similar to C group (**Figures 3B,D, E**). *Roseburia* was also significantly reduced in P-N group compared to C group, but its relative abundance was significantly higher in P-Bb group than C group (**Figure 3C**). And the *Clostridium*, which had low relative abundance in group C, was significantly higher in group P-N than in group P-Bb (**Figure 3F**).

There was no increase in adverse events with postoperative probiotics administration compared with placebo. In addition to the symptoms associated with the primary clinical endpoints described above, there was one case of hematochezia and one case of insomnia in the P-Bb group. The P-N group had one case of more severe diarrhea and two cases of hematochezia. The researchers concluded that the above symptoms may not be related to MH-02 intake and that oral MH-02 is considered to be very safe. No other serious adverse events or deaths occurred.

## Discussion

This prospective study showed efficacy of MH-02 in patients receiving resection of multiple colorectal polyps. Compared to placebo, MH-02 showed no significant improvement in postoperative symptoms, and among single symptoms, only difficult defecation was significantly improved. However, MH-02 allowed faster recovery of bowel function and reduced the frequency of laxative use. High-throughput analysis showed that MH-02 can help restore the diversity of intestinal microbiota, and increased the relative abundance of *Bifidobacterium*, *Roseburia*, *Gemmiger*, *Blautia* and *Ruminococcus*, while reduced the relative abundance of *Clostridium*, and the alteration of these bacteria was beneficial to health. Finally, administration of MH-02 is considered to have high acceptance and safety.

Colorectal polyps are a common and potentially dangerous intestinal disease that requires early endoscopic surgical resection (1, 4). The procedure is minimally invasive and the incidence of serious complications such as bleeding and perforation is less than 1% (22), however, minor complications such as abdominal pain and bloating occur in more than 30% of cases within 7 days after resection (5). In addition, difficult defecation is also a common symptom after surgery. Difficult defecation is the most common symptom in patients with constipation, with a prevalence of 68% in functional constipation (23). Patients are prone to constipation after gastrointestinal surgery (24), probably due to bowel preparation and surgical stress. A previous study showed that the administration of Lactobacillus acidophilus and Bifidobacterium lactis did not reduce abdominal pain and bloating in patients after colonoscopy, but the results were reversed in a subgroup analysis of preoperative symptomatic patients (21). Similarly, another study revealed that Bacillus subtilis and Streptococcus faecium, started 2 weeks before surgery, were effective in improving the onset of postcolonoscopy symptoms in patients with preoperative constipation, but the results were negative in the preoperative asymptomatic group (25). In this study, MH-02 provided no significant improvement in symptoms within 7 days after resection of multiple colorectal polyps, and performed a therapeutic effect only in difficult defecation when single symptom analysis was performed. Multiple regression analysis revealed that polyp size and preoperative symptoms were factors influencing the efficacy of MH-02 in improving postoperative symptoms. Bowel dysfunction can be commonly seen after colonoscopy (26), and it takes several days to return to normal bowel habits. Postoperative constipation is the major reason affecting the recovery of bowel function. A previous study showed little effect of probiotics on intestinal function, with only subgroups showing such positive results (21). In this study, MH-02 significantly reduced the time to recovery of bowel function and reduced the incidence and severity of constipation in patients. Overall, MH-02 provided an adjuvant therapeutic effect after resection of colorectal polyps, especially in the recovery of bowel function in patients, and there may be group differences in the therapeutic effect.

Resection of colorectal polyps may result in a severe dysbiosis of the intestinal microbiota. Bowel preparation is a key step in the gastrointestinal surgery, and our previous study showed that oral *Bifidobacterium* after bowel preparation significantly increased the diversity of the intestinal microbiota, and reduced the relative abundance of pathogenic *Acinetobacter*, while enriching the relative abundance of *Roseburia* and *Faecalibacterium* (10), suggested a critical role for *Bifidobacterium* in the recovery of intestinal microbiota. In this study, MH-02 significantly increased the α­diversity and β-diversity of the intestinal microbiota of postoperative patients. In addition, the clustering heat map showed that MH-02 could cause changes in the abundance of a variety of bacteria. *Bifidobacterium*, *Faecalibacterium*, *Dorea*, *Roseburia*, *Gemmiger*, *Blautia*, and *Ruminococcus* had higher abundance in patients taking MH-02, and these bacteria always play an active role in intestinal inflammation, immunity and tumor. *Bifidobacterium*, the main commensal flora of the intestine, has a high and stable relative abundance in the intestine of healthy adults (14), and can reduce inflammation after intestinal surgery and suppress tumors (15, 17). *Faecalibacterium* and *Roseburia* are the most important butyrate-producing bacteria in the human colon, and their presence may be associated with a reduced risk of chronic inflammation of the intestine (27, 28). Butyrate is a product of dietary fiber fermentation by bacteria and may exert tumor suppressive effects *via* pathways such as Gpr109a-butyrate signaling (29). *Dorea* is the main gas-producing bacterium in the human intestine and may be associated with irritable bowel syndrome (30). *Ruminococcus*, one of the first stomach bacteria identified, has an important role in metabolism and has also been suggested to exert beneficial effects such as stabilizing the intestinal barrier and reducing the risk of colorectal cancer (31). The abundance of *Ruminococcus* and *Gemmiger* is negatively correlated with intestinal inflammation (32, 33). *Blautia* is widely present in the mammalian gut and considered to be a beneficial bacterium that plays a role in metabolic diseases, inflammatory diseases and biotransformation (34). Moreover, a study reported reduced abundance of *Blautia* in mucosal adherent microorganisms in patients with colorectal cancer (35). *Dorea*, *Roseburia*, *Gemmiger*, *Blautia*, and *Ruminococcus* were at the same taxonomic level in the clustering tree, indicating that these beneficial Bacteria had similar abundance in the samples and may have synergistic effects. In contrast, *Megamonas* and *Clostridium* were present in higher abundance in patients taking placebo after surgery. The abundance of *Megamonas* is significantly higher in Asian colorectal cancer population (36). *Clostridium* can produce exotoxins that become the cause of intestinal diseases, and it has been shown that specific species of *Clostridium* such as Clostridium difficile are closely associated with the development of colorectal cancer (37). However, age is also an important factor affecting the intestinal microbiota. In this experiment, the patients’ ages were concentrated between 50 and 70 years, and our samples were collected within 1 week after the patients’ surgery, when the intestinal microbiota disorder had not fully recovered and the probiotic intervention was the main influencing factor for this recovery process. To sum up, MH-02 can help restore intestinal microbiota balance and may provide evidence to further explain the mechanism of the effect of probiotics in patients with resection of colorectal polyps.

It is reported that 20-50% of patients with colorectal polyps are at risk of postoperative recurrence (6). Previous studies have suggested that the intestinal microbiota may be involved in the recurrence of colorectal polyps and even colorectal cancer after surgery, and that modulating the composition of the intestinal microbiota may be able to reduce recurrence outcomes (12, 38). In the present study, MH-02 altered the composition of the intestinal microbiota and changed the bacteria such as *Bifidobacterium*, *Ruminococcus*, *Roseburia*, *Blautia*, *Gemmiger* and *Clostridium* in a healthy direction. Longer time probiotics intervention is needed to determine whether it has the effect on stably altering the intestinal microbiota composition and influencing the outcome of colorectal polyp in the future.

The limitation of this study is that the homogeneity of preoperative symptoms of patients was not controlled. Preoperative symptoms are an important factor in the efficacy of probiotics, it is more appropriate to investigate people with homogeneous preoperative symptoms for relevant trials. Another limitation is that although we analyzed clinical symptoms, bowel function and changes in intestinal microbiota, direct evidence of the link among them was not explored. In addition, the sample size of this trial was estimated based on postoperative symptom scores, which are closely related to patients’ preoperative symptoms, and the sample size required for the trial varies among different preoperative symptom populations, which may be the main reason for the small sample size estimate of this trial.

The strength of this trial is that we conducted a rigorous trial design, including strict inclusion and exclusion criteria, as well as detailed symptom scoring criteria. And the assessment was performed by the same highly trained person, which reduced other potential sources of variability. We also attempted to explain the possible mechanisms at the microbial level and succeeded in identifying some clue bacteria.

In conclusion, MH-02 showed efficacy in patients after resection of colorectal polyps, especially in the reduction of difficult defecation and restoration of bowel function. Meanwhile, MH-02 could help to restore the balance of intestinal microbiota, and the alteration of some bacteria may provide help to further explain its mechanism. Future studies should focus on the role of probiotics in different populations and need to further explore the mechanisms. Moreover, the effect of long-term probiotic intervention on the outcome of colorectal polyp recurrence could be investigated.

## Data Availability Statement

The datasets presented in this study can be found in online repositories. The names of the repository/repositories and accession number(s) can be found in the article/**Supplementary Material**.

## Ethics Statement

The studies involving human participants were reviewed and approved by the Ethics Committee of the First Hospital of Nanchang. The patients/participants provided their written informed consent to participate in this study.

## Author Contributions

NF: conceptualization, funding acquisition, supervision, writing review, and editing. TC: conceptualization, funding acquisition, writing review, editing, and assistance with formal analysis of data. HL: experimentation, references, original draft. KZ: experimental assistance, data formal analysis assistance. PL: experimental assistance. XX: editing assistance. YZ: experimental assistance. LG: editing assistance. LY: editing assistance. BL: experimental assistance. All authors contributed to the article and approved the submitted version.

## Funding

This study was supported by National Natural Science Foundation of China (No. 8166100442, 82060638), Double thousand plan of Jiangxi Province (High-End Talents Project of scientific and technological innovation), Academic and Technical Leaders of Major Disciplines in Jiangxi Province (S2019RCDT2B0168), Double Hundred Plan for High-level Scientific and Technological Talents in Nanchang City (2020137) and Postgraduate Innovation Special Fund Project in Jiangxi Province (YC2021-S213).

## Conflict of Interest

The authors declare that the research was conducted in the absence of any commercial or financial relationships that could be construed as a potential conflict of interest.

## Publisher’s Note

All claims expressed in this article are solely those of the authors and do not necessarily represent those of their affiliated organizations, or those of the publisher, the editors and the reviewers. Any product that may be evaluated in this article, or claim that may be made by its manufacturer, is not guaranteed or endorsed by the publisher.
