# Supplementary material for: Improvement Effect of Bifidobacterium animalis subsp. lactis MH-02 in Patients Receiving Resection of Colorectal Polyps: A Randomized, Double-Blind, Placebo-Controlled Trial
Source: Front Immunol. 2022 Jun 27;13:940500. doi: 10.3389/fimmu.2022.940500 (PMC9271559; doi:10.3389/fimmu.2022.940500)
Supplement: Supplementary file 1 [file Table_1.docx]

|  | **P-Bb(n=48)** | **P-N(n=52)** |
| --- | --- | --- |
| Pain | 12(3ab, 2abd, 2ac, 1ae) | 11(4ab, 1abd, 2ac) |
| Bloating | 16(3ab, 2abd, 5bd) | 18(4ab, 1abd, 7bd) |
| Abnormal bowel habits | 20(5c, 13d, 2e) | 22(4c, 17d, 1e) |
| Other cause | 3 | 2 |
| symptom absent | 12 | 14 |

**Supplementary Table 1** **|** Preoperative symptoms

Some patients had multiple symptoms before surgery. a=Pain; b= Bloating; c= Frequent defecation; d= Difficult defecation; e= hematochezia.
